# Multiplex quadruple bioluminescent assay system

**DOI:** 10.1038/s41598-022-20468-1

**Published:** 2022-10-19

**Authors:** Genta Kamiya, Nobuo Kitada, Shojiro Maki, Sung Bae Kim

**Affiliations:** 1 Department of Engineering Science, Graduate School of Informatics and Engineering, The University of Electro-Communications, Chofu, Tokyo 182-8585 Japan; 2 Research Institute for Environmental Management Technology, National Institute of Advanced Industrial Science and Technology (AIST), 16-1 Onogawa, Tsukuba, 305-8569 Japan

**Keywords:** Biological techniques, Biotechnology, Chemical biology, Endocrinology, Analytical chemistry, Biochemistry, Chemical biology, Organic chemistry, Photochemistry

## Abstract

Bioluminescence (BL) is unique cold body radiation of light, generated by luciferin–luciferase reactions and commonly used in various bioassays and molecular imaging. However, most of the peak emissions of BL populate the blue-yellow region and have broad spectral bandwidths and thus superimpose each other, causing optical cross-leakages in multiplex assays. This study synthesized a new series of coelenterazine (CTZ) analogues, named K-series, that selectively illuminates marine luciferases with unique, blue-shifted spectral properties. The optical property and specificity of the K-series CTZ analogues were characterized by marine luciferases, with K2 and K5 found to specifically luminesce with ALuc- and RLuc-series marine luciferases, respectively. The results confirmed that the luciferase specificity and color variation of the CTZ analogues minimize the cross-leakages of BL signals and enable high-throughput screening of specific ligands in the mixture. The specificity and color variation of the substrates were further tailored to marine luciferases (or single-chain bioluminescent probes) to create a multiplex quadruple assay system with four integrated, single-chain bioluminescent probes, with each probe designed to selectively luminesce only with its specific ligand (first authentication) and a specific CTZ analogue (second authentication). This unique multiplex quadruple bioluminescent assay system is an efficient optical platform for specific and high-throughput imaging of multiple optical markers in bioassays without optical cross-leakages.

## Introduction

Bioluminescence (BL) is unique cold body radiation of light, generated by luciferin–luciferase reactions within living organisms. Thus far, BL is used as an indispensable analytical tool in various fields of science and technology, such as in methods of measuring ATP, Ca^2+^, and superoxide anions^1^.

BL has distinct advantages when used in bioassays and molecular imaging, including low background intensity, a high signal-to-noise ratio (S/N), no external light source, a wide dynamic range, and biocompatibility in living subjects^2,3^. The BL platform also has versatile applications in the molecular design of imaging probes; for example, luciferase can be split into two fragments and reconstituted for imaging protein–protein interactions (PPIs)^4^, and it can link to fluorescent proteins (FP) to exert bioluminescence resonance energy transfer (BRET) imaging^5,6^.

However, BL has a critical issue that needs to be addressed on use in multiplex imaging. BLs have diverse colors ranging from blue to red, but most of the peak emissions populate the blue-yellow region^3,7^. Further, the spectra have broad bandwidths and thus superimpose each other. This optical cross-leakage severely impairs multiplex imaging.

As a result, BL systems with two or more multiple luciferase reporters conventionally make use of quenchers, filters, and/or deconvolution schemes to discriminate the multiple BL signals with minimal optical cross-leakage^8,9^. Alternatively, instead of pursuing perfect signal unmixing, some researchers have made use of the patterns of substrate use, i.e., “fingerprints,” to discriminate combinations of multiple luciferases^10^. However, this cannot be considered as the fundamental solution to the issue.

Biological systems are complex. Hence single biological measurements yield only limited information. Additional assays must be performed to expand information content, but this requires protracted assay development, time, and/or expanse. Moreover, independently performed assays may not always be appropriately controlled, making comparative analysis across different experimental variables difficult^11^. Multiplex assay systems seek to address these limitations by measuring multiple reporters from a single screening unit simultaneously^8^ and thus can provide collective information with high fidelity, reflecting the complex context of biological systems.

The present research, therefore, aims to investigate a fundamental breakthrough to solve the problem in the analytical use of multiple BL. First, the chemistry of luciferase–luciferin reactions was reviewed. Luciferin has been recited to be its energy source, acting as “luminophore” in the BL-emitting reaction^1^. Thus, chemical modification of luciferins was considered as the fundamental solution to exerting specificity to each BL reporters and preventing optical cross-leakages among the multiple BL reporters.

We have previously reported two coelenterazine (CTZ) analogues that are specific to *Renilla* luciferase 8.6-535 (RLuc86) and artificial luciferase 16 (ALuc16), respectively^12^. However, this study validated the utility within dual luciferases, and the two luciferases have a large discrepancy in optical intensities when used in practical dual assays.

In the present work, a new series of CTZ analogues (consecutively named K1–6) was synthesized with the modified C-6 and C-8 positions, specifically: (i) by elimination of the π-electron conjugation or extension of the π-electron conjugation through replacement of the benzyl group with a phenyl group at the C-8 position of the imidazopyrazinone backbone and (ii) by introduction of a dimethylaminophenyl group or a bulky benzodioxane group to the C-6 or C8 positions of the backbone. This chemical structural modification was done with the speculation of an exceptional luciferase specificity and spectral variations.

The optical properties of K1–6 were characterized with respect to the luciferase specificity, chemical stability (autoluminescence), spectral shifts, and bioanalytical utilities. The practical bioanalytical advantages of the K-series CTZ analogues were found to simultaneously determine four ligands with a multiplex quadruple bioassay system. The present research is thus an important addition to bioassay and molecular imaging studies in chemistry and biology.

## Experimental procedure

### Design and organic synthesis of new CTZ analogues

**Figure 1:**
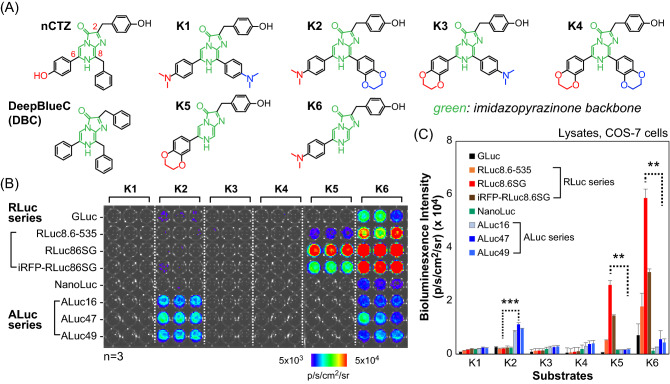
(**A**) The specific chemical structures of the synthetic luciferins, K1–K6, as well as conventional native coelenterazine (nCTZ) and DeepBlueC (DBC). The characteristic functional groups are highlighted in red; the imidazopyrazinone backbone is shown in green. (**B**) The optical intensity matrix between all the synthetic luciferins and major marine luciferases (n = 3). The left end annotation indicates each luciferase that is transiently expressed in mammalian COS-7 cells cultured in each row of a 96-well black frame microplate. (**C**) The absolute BL intensities of the synthetic luciferins according to various marine luciferases. The p-values (Student's *t* test) are ***≤ 0.001 and **≤ 0.01. All *p* values are for comparison of the data sets of RLuc86SG and ALuc47 for each substrate.

In the present study, a new series of CTZ analogues was synthesized with modification of the C-8 and C-6 positions (Fig. 1A). The benzyl group of native CTZ (nCTZ) was replaced with a phenyl group at the C-8 position. Further, a dimethylaminophenyl or benzodioxane group was introduced in the C-6 or C-8 groups. The newly synthesized substrates were named Kamiya 1–6 (K1–6) after the first author of this work. The specific synthesis method of the present K-series CTZ analogues is described in detail in Experimental Procedure S1.

### Luciferase specificity of the K-series CTZ analogues

The luciferase specificity of the K-series CTZ analogues was characterized with various marine luciferases (Fig. 1B).

COS-7 cells derived from African green monkey kidney (Cell Bank, Riken BRC, Japan) were grown in 6-well microplates with a culture medium, i.e., Dulbecco’s Modified Eagle Medium (DMEM), supplemented with 10% (v/v) fetal bovine serum (FBS) and 1% (v/v) penicillin and streptomycin (P/S) mixture. When 70% confluency was reached, the cells in each well were transiently transfected with a pcDNA3.1(+)plasmid encoding one of the following marine luciferases by using the lipofection reagent TransIT (Mirus): (i) *Gaussia* luciferase (GLuc), (ii) *Renilla* luciferase 8.6-535 (RLuc86), (iii) *Renilla* luciferase 8.6-535SG (RLuc86SG), (iv) iRFP-RLuc86SG, (v) NanoLuc, (vi) ALuc16, (vii) ALuc47, and (viii) ALuc49.

The cells were incubated for one day, trypsinized, subcultured in a 96-well black frame microplate (10^4^ cells per well), and then incubated overnight. Then, the culture media were thoroughly eliminated, and the remaining cells in each well were lysed with 40 μL of lysis buffer (Promega) for 20 min. The microplate containing the cell lysates was kept at room temperature (RT) before BL measurement.

On the other hand, all the K-series CTZ analogues were first dissolved with 100% methanol to 100 μM (stock solution) and further diluted with phosphate-buffered saline (PBS) to 5 μM. The final methanol concentration is less than 1%. The diluted CTZ analogues were aliquoted in each well of a fresh 96-well microplate. Then, 40 μL of the diluted CTZ analogues per well were simultaneously injected into each well of the microplate containing the cell lysates by using a 12-channel micropipette. The corresponding BL intensities were immediately determined with an IVIS imaging system (Xenogen, USA) and analyzed with the Living Image version 5.7 software.

**Figure 2:**
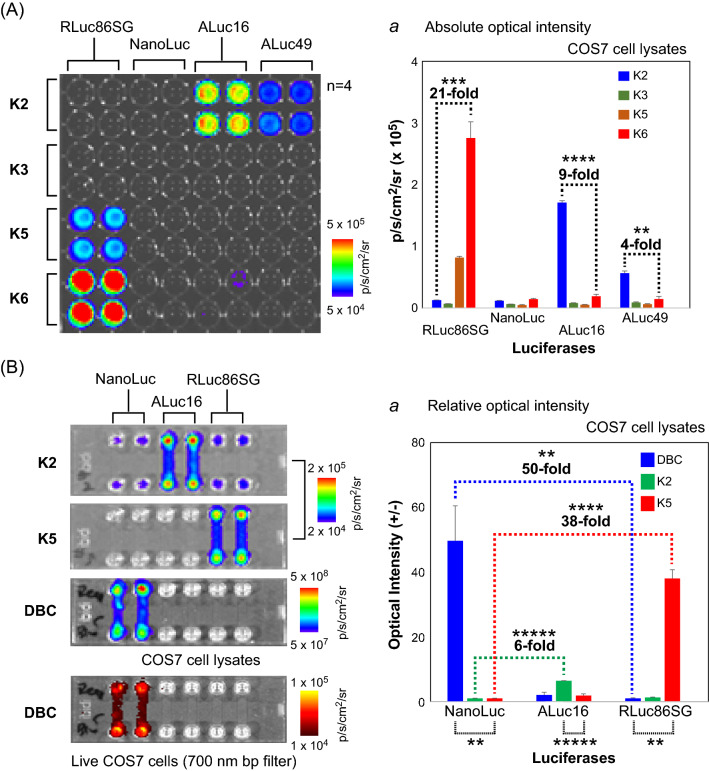
(**A**) The optical intensity matrix between selected synthetic luciferins and marine luciferases. Inset *a* denotes the corresponding absolute intensities. The numbers on the bars indicate the fold intensities of K2 or K6, compared with the opposite. The *p* values (Student's t test) are **** ≤ 0.0001 and ** ≤ 0.01. (**B**) The optical specificity of the synthetic luciferins to marine luciferases, expressed in COS-7 cells cultured in microslides. Inset *b* shows the corresponding fold intensities. The numbers on the bars indicate the fold intensities, compared with the minimal values. The *p* values (Student's *t* test) are *****≤ 0.00001, ****≤ 0.0001, and **≤ 0.01.

The luciferase specificity was further highlighted with selected pairs of K-series CTZ analogues and marine luciferases (Fig. 2A). The K-series CTZ analogues and the COS-7 cells transiently expressing RLuc86SG, NanoLuc, ALuc16, or ALuc49 were prepared according to the protocol shown in Fig. 1B. The corresponding BL intensities were determined by using the method presented in Fig. 1B.

The corresponding live cell images were determined with 6-channel microslides (ibidi, Germany) (Fig. 2B). COS-7 cells prepared with the protocol in Fig. 1B were subcultured in each channel of the microslides, with the cells containing each marine luciferase deployed to every two channels. The culture media in the channels were completely eliminated, and the channels were simultaneously filled with 60 μL of the diluted CTZ analogue, i.e., K2, K5, or DeepBlueC (DBC), by using a multichannel micropipette. The subsequent BL images were determined with the IVIS imaging system (Xenogen, USA) and analyzed with the Living Image version 4.7 software.

Further, the cells in the microslides were lysed with the protocol shown in Fig. 1B, after complete removal of the culture media. The BL intensities were determined and analyzed by applying the method shown in Fig. 2A.

### Quantitative relationship among the cells stably expressing the marine luciferases

**Figure 3:**
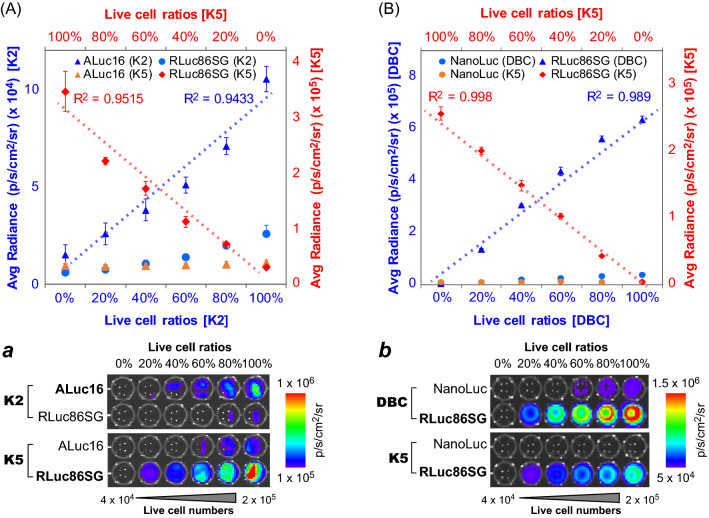
(**A**) Determination of the quantitative relationship between ALuc16 and RLuc86SG in the presence of the specific substrate K2 or K5. The defined amount of live COS-7 cells expressing ALuc16 were mixed with the corresponding amount of those expressing RLuc86SG at different ratios totaling 100%. Inset (a) shows the corresponding BL images of the live cells expressing ALuc16 or RLuc86SG. (**B**) Determination of the quantitative relationship between NLuc and RLuc86SG in the presence of the specific substrate DBC or K5. The defined amount of live COS-7 cells expressing NLuc were mixed with the corresponding amount of those expressing RLuc86SG at different ratios totaling 100%. The consequent BL intensities were determined after simultaneous injection of DBC or K5. Inset (b) shows the corresponding BL images of the live cells.

The quantitative relationships were determined among the live cells stably expressing RLuc86SG, ALuc16, or NanoLuc (Fig. 3).

Initially, the COS-7 cells were transiently transfected with the pcDNA3.1(+) vector encoding RLuc86SG, ALuc16, or NanoLuc. The cells stably expressing each luciferase were then screened by serial passage and long-term culture of the cells in the culture medium containing 0.5 mg/mL Geneticin (G418) as the final concentration.

The cells stably expressing RLuc86SG, ALuc16, or NanoLuc were harvested through trypsinization and centrifuge and then resuspended in PBS to reach 1 × 10^3^ cells per μL. The resuspended cells (100%) were consecutively diluted with PBS to 80%, 60%, 40%, 20%, and 0%. Each dilution (20 μL) was aliquoted into each well of a 96-well black frame microplate in order.

The cells in the wells were simultaneously mixed with 40 μL of the diluted K2, K5, or DBC solutions (5 μM) by using a 12-channel micropipette. The corresponding BL images were determined through the IVIS imaging system and analyzed with the Living Image version 5.7 software.

### Autoluminescence of the K-series CTZ analogues

The chemical stability of the K-series CTZ analogues was examined with respect to the autoluminescence in serum samples (Fig. S1).

FBS (100%) was consecutively diluted with PBS, and four different serum solutions were prepared, i.e., 100%, 40%, 20%, and 0%. The stock solutions of the K-series CTZ analogues were separately diluted with PBS to 20 μM.

The serum solutions (40 μL) were deployed in each well of a 96-well black frame microplate and simultaneously mixed with the same volume of the K-series CTZ analogues by using a multichannel micropipette. The final FBS ratios were 50%, 20%, 10%, and 0%, respectively. The microplate was immediately transferred to the chamber of the IVIS imaging system, and the corresponding BL images were determined and analyzed with the Living Image version 5.7 software.

### BL spectra of new CTZ analogues

**Figure 4:**
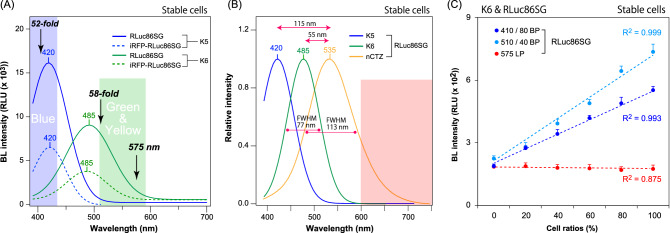
(**A**) The absolute BL spectra of selected synthetic luciferins, K5 and K6, in the presence of RLuc86SG or iRFP-RLuc86SG. The arrows with annotations “52-fold” and “58-fold” highlight the fold intensities of the RLuc86SG―K5 and RLuc86SG―K6 pair at 393 and 523 nm, respectively. (**B**) Normalized BL spectra of selected CTZs, K5, K6, and nCTZ in the presence of RLuc86SG. FWHM refers to the full width at half maximum of the BL spectra. The red shadow highlights the red region in the visible light spectrum. (**C**) Determination of the quantitative relationship between the BL intensities of RLuc86SG in the violet (410 nm), green (510 nm) or the orange and red (570 nm and longer) regions in the presence of K6. The defined amount of live COS-7 cells expressing RLuc86SG were mixed with the corresponding amount of PBS at different ratios totaling 100%. The corresponding BL intensities were determined with a 410-nm band-pass or 570-nm long-pass filter.

Because RLuc86SG showed strong BL with K5 and K6, the corresponding BL spectra were examined (Fig. 4). The COS-7 cells stably expressing RLuc86SG or iRFP-RLuc86SG were established, as described in Fig. 3.

The two types of COS-7 cells were cultured separately in a 6-well microplate and then harvested by trypsinization and centrifuge. The supernatant was completely eliminated, and the remaining cells were resuspended in PBS to 10^3^ cells/μL. The cell suspensions (20 μL) were aliquoted to PCR tubes. Then, 40 μL of the K5 or K6 solution was added to the PCR tube, which was immediately mounted in a spectrophotometer (AB-1850, ATTO). The corresponding BL spectra were determined with the high sensitivity mode and 1-min integration time.

The consequent BL spectra were smoothed with the algorithm “box” of Igor Pro 7.08 and analyzed with the same software.

### Creation of two cDNA constructs encoding new single-chain bioluminescent probes for assaying androgen and rapamycin

**Figure 5:**
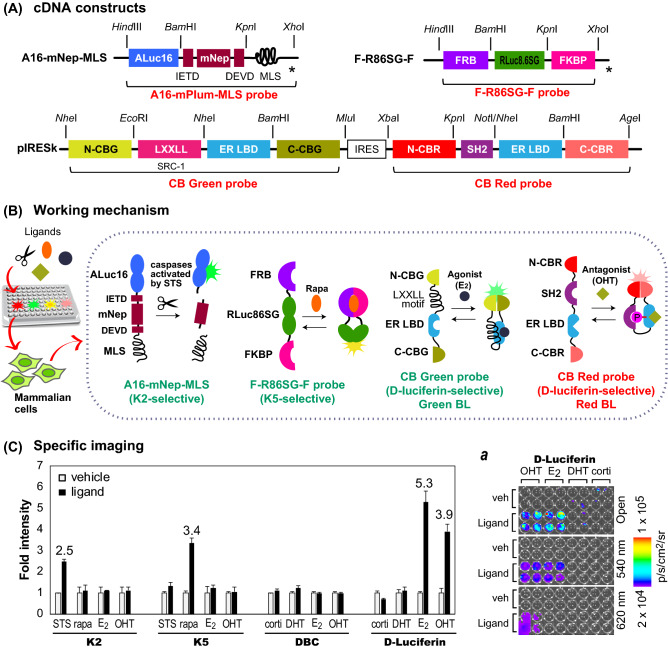
(**A**) Schematic diagram of the cDNA constructs encoded in mammalian expression vectors. The upper panel shows the cDNA constructs encoding A16-mNep-MLS and iRFP-R86SG-NLS; the lower panel shows the cDNA constructs encoding the CB green and CB red probes. (**B**) Four different working mechanisms of four single-chain bioluminescent probes in the same microtube according to their specific ligands. Each probe is designed to change its conformation by a specific ligand and to luminesce only with its specific substrate. (**C**) Simultaneous determination of the fold intensities of the four probes in the presence or absence of their specific ligands and substrates (n = 3). The numbers on the bars indicate the fold intensities. Inset (a) shows the corresponding BL images.

Genetically encoded single-chain bioluminescent probes for assaying endogenous androgen and rapamycin were created (Fig. 5). The corresponding cDNA constructs were made according to the following procedure.

For the single-chain probe for androgen assaying, two cDNA segments encoding the FKBP12-rapamycin binding domain of mTOR (FRB) and the FK506 binding protein (FKBP) were generated through a polymerase chain reaction (PCR) by using corresponding primers to introduce unique restriction sites, i.e., *Hind*III/*BamH*I and *Kpn*I/*Xho*I at the 5′ and 3′ ends, respectively. Separately, a cDNA segment encoding RLuc86SG was created through a PCR by using corresponding primers to introduce *BamH*I/*Kpn*I sites at the 5′ and 3′ ends. The PCR products were purified by electrophoresis. The three purified segments were double digested by the corresponding restriction enzymes, ligated with a ligation kit, and finally subcloned into the pcDNA3.1(+) vector for mammalian expression. This probe after expression was named F-R86SG-F.

Similarly, three sets of cDNA segments encoding N- and C-terminal fragments of RLuc86SG were made by a PCR to introduce *Hind*III/*Kpn*I and *BamH*I/*Xho*I sites at the 5′ and 3′ ends, respectively. The only difference between the three sets were the fragmentation points: the first set encodes 1–222 aa and 223–311 aa of RLuc86SG, the second set encodes 1–229 aa and 230–311 aa of RLuc86SG, and the third set encodes 1–234 aa and 235–311 aa of RLuc86SG.

On the other hand, the chimera cDNA segment encoding the ligand binding domain of the human androgen receptor (AR LBD) and the leucine-rich motif were obtained from our previous study^13^. Because the chimera cDNA segment already comprises *Kpn*I and *BamH*I sites at the 5′ and 3′ ends, respectively, the chimera was directly used without additional PCR. All the part segments were double digested by the corresponding restriction enzymes, ligated, and finally subcloned into the pcDNA3.1(+) vector for mammalian expression. This probe after expression was named SiAR86.

The fidelity of the cDNA constructs was confirmed by DNA sequencing.

### Bioluminescent assays for rapamycin by using F-R86SG-F

COS-7 cells grown in a 6-well microplate were transiently transfected with the pcDNA3.1(+) vector encoding F-R86SG-F (Suppl. Figure 2). After one day of incubation, the cells were harvested by trypsinization and centrifuge and finally subcultured on 6-channel microslides, which were then incubated overnight in a 5% (v/v) CO_2_ incubator.

The three left channels were then stimulated with the culture medium carrying vehicle (0.1% (v/v) DMSO), whereas the three right channels were prepared with the same culture medium containing 10^–6^ M rapamycin. The microslide was stored in a CO_2_ incubator for 5 h.

After complete decantation of the media, the channels of the microslide were simultaneously filled with 60 μL of the diluted K5 or K6 solutions (5 μM) by using a multichannel micropipette. The microslides were placed in the chamber of the IVIS system, and the corresponding BL images were determined and analyzed with the Living Image version 4.7 software.

### Construction of genetically encoded multiplex quadruple BL assay system

A genetically encoded quadruple BL assay system with mammalian expression vectors encoding four single-chain bioluminescent probes was constructed (Fig. 5).

The unique cDNA constructs encoding click beetle luciferase green (CB green) probe and click beetle luciferase red (CB red) probe were obtained from our previous study^14^ and deployed in the two multicloning sites (MCS) of a pIRES vector (two probes per vector), as shown in Fig. 5A. The other two probes are made of two distinctive marine luciferases, the designs of which were inspired by our previous studies^15,16^: ALuc16 for A16-mPlum-MLS, RLuc86SG for F-R86SG-F. Among the cDNA constructs encoding the four probes, those encoding A16-mPlum-MLS and F-R86SG-F were developed in this study through modifying the cDNA constructs from our previous studies^15,16^.

The fidelity of the cDNA sequences was confirmed through DNA sequencing.

### Simultaneous determination of four different ligands by a multiplex quadruple bioluminescent assay system

The genetically encoded multiplex quadruple bioluminescent assay system was characterized if it can simultaneously determine four different ligands (Fig. 5).

COS-7 cells grown in a 10-cm culture dish were cotransfected with the three vectors for expressing four probes, i.e., A16-mPlum-MLS, F-R86SG-F, CB green probe, and CB red probe, by using the lipofection reagent TransIT-LT1 (Mirus). The cells were then incubated for one day in a 5% (v/v) CO_2_ incubator, harvested by trypsinization and centrifuge, and then subcultured in the wells of a 96-well black frame microplate.

The wells of the microplates were conceptionally divided into four sections, with the wells of each section further subdivided into four subsections. The cells in each subsection were stimulated with vehicle (0.1% DMSO), 10^–6^ M staurosporine (STS), 10^–6^ M rapamycin (rapa), 10^–6^ M 17β-estradiol (E_2_), or 10^–6^ M 4-hydroxy-tamoxifen (OHT). or 10^–6^ M cortisol (corti), 10^–6^ M dihydrotestosterone (DHT), 10^–6^ M 17β-estradiol (E_2_), or 10^–6^ M OHT dissolved in the culture media, where the cells were stimulated for 1 h with the steroids; for 5 min with STS; for 5 h with rapamycin. The culture media in the wells were then completely removed, and the cells were lysed with 40 μL of a lysis buffer (Promega) per well and incubated in RT for 15 min.

The ligands were chosen because of the following reason: STS is a representative apoptosis inducer, which activates caspases in mammalian cells. The activated caspases release ALuc16 through dissection of the specific substrate sequences, IETD and DEVD inside A16-mNep-MLS. Free ALuc16 is much brighter than the originally fused one. We named it “bioluminescent capsule” in our precedent study^15^. Rapamycin is a model ligand to exert FRB–FKBP binding in F-R86SG-F probe. Rapamycin-driven intramolecular FRB–FKBP binding append molecular strain to the sandwiched RLuc86SG. Strained RLuc86SG is much brighter than relaxed RLuc86SG. We named this type of probe “molecular strain probe” in our previous studies^16,17^. E_2_ and OHT are the agonist and antagonist of estrogen receptor (ER), respectively. The CB green and red probes are sensitive to E_2_ and OHT, respectively. The CB green and red probes luminesce green and red BL in the presence of D-luciferin. We named it “multicolor imaging probes” in our previous study^14^.

The wells in each section of the microplate were simultaneously injected with 40 μL of K2, K5, DBC (all dissolved in PBS), or D-Luciferin dissolved in Bright-Glo assay buffer (Promega). The corresponding BL images were determined with a microplate reader (TriStar^2^ S LB942, Berthold) or the IVIS system and analyzed with the Living Image version 4.7 software.

## Results

### Among the new CTZ analogues, K2 and K5 are highly specific to ALucs and RLucs, respectively

The luciferase specificity of the K-series CTZ analogues with various marine luciferases was investigated (Fig. 1B,C). The quantitative analysis showed that K2 was highly selective only to ALuc series luciferases, whereas K5 was specific only to RLuc series luciferases. K6 in particular emitted the strongest BL with RLuc86SG. The absolute BL intensities were strongest for K6, K5, and K2 in that order. Meanwhile, K1, K3, and K4 failed to emit detectable levels of BL intensities with the marine luciferases.

The luciferase specificity was further highlighted with selected substrates and luciferases (Fig. 2). As shown in Fig. 2A, K2 was highly selective to ALuc series luciferases, whereas K5 and K6 were specific only to RLuc series luciferases. For instance, the K6–RLuc86SG pair luminesced 21-fold stronger than the K2–RLuc86SG pair, whereas the K2–ALuc16 pair was optically ninefold stronger than the K6–ALuc16 pair. On the other hand, all the K-series substrates did not luminesce with NanoLuc. The optical intensities of K2–ALuc16 pair and K2–ALuc49 pair were almost equivalent in Fig. 1C, whereas those intensities in Fig. 2A are more variant. We understand that this discordance may be caused by the variance in the experimental conditions between the batches including the gene transfection efficiency and the relative chemical stability of the substrates.

The luciferase specificity of K2 and K5 was found to be preserved even in live animal cells, such as COS-7 cells, as shown in the BL image in Fig. 2B. K2 luminesced sixfold brighter with ALuc16 than with NanoLuc, and K5 showed 38-fold stronger BL intensities with RLuc86SG than with NanoLuc. In contrast, DBC was 50-fold brighter with NanoLuc, compared with RLuc86SG. The near infrared (NIR) image of DBC–NanoLuc pair is originated from the tailed BL, although most of the light is in blue. The K-series CTZ analogues have no apparent cytotoxicity during the cell-based assays.

The overall results indicate that the three reporters (ALuc16, RLuc86SG, and NanoLuc) can be specifically quantified with K2, K5, or DBC, respectively, with minimal optical cross-leakages in animal cell models.

The quantitative relationships among the live cells stably expressing each marine luciferase were determined in the presence of K2, K5, or DBC (Fig. 3).

The correlation graph in Fig. 3A confirms that the live cells stably expressing RLuc86SG and ALuc16 were selectively illuminated by K5 (red) and K2 (blue), respectively. For example, the 20%-content live cells containing RLuc86SG showed 5.9 × 10^4^ p/s/cm^2^/sr with K5, whereas the same live cells containing ALuc16 marked merely 2.2 × 10^3^ p/s/cm^2^/sr with the same K5, almost equivalent to that of the background. This feature was completely reversed with K2.

The correlation coefficients between the BL intensities and the live cell ratios were 0.9515 for the RLuc86SG–K5 pair and 0.9433 for the ALuc16–K2 pair, respectively. This strong linearity indicates that the BL intensities are proportional to the stable cell numbers.

The correlation graph in Fig. 3B describes a slightly different feature from that in Fig. 3A. It is commonly true that K5 specifically illuminates the live cells containing RLuc86SG. However, DBC did not significantly luminesce with the cells stably expressing NanoLuc, compared with the cells with RLuc86SG. This is in contrast to the results obtained with the cells transiently expressing NanoLuc. The live cells stably expressing NanoLuc were presumed to express a poor level of NanoLuc and thus to luminesce less, regardless of the selectivity. As quantitatively shown in Fig. 3B, The RLuc86SG-DBC is approximately twofold brighter than RLuc86SG-K5 combination.

Based on the overall results, the three marine luciferases have a quantitative relationship with each other, as well as specificity to K2, K5, or DBC.

### K3 and K6 emit considerable autoluminescence in serum samples

The chemical stability of the K-series CTZ analogues in biological samples is important in their application to bioassays and animal imaging. Thus, the autoluminescence of the CTZ analogues in serum samples was determined (Fig. S1).

The results showed that K6 strongly autoluminesced in serum samples in the whole ratio range of FBS. K6 reached the highest autoluminescence level at 2.4 × 10^6^ p/s/cm^2^/sr (average radiance), followed by K3. The other CTZ analogues, i.e., K1, K2, K4, and K5, did not exert significant autoluminescence in the whole ratio range of FBS. Their highest autoluminescence levels in the severe conditions were about 1 × 10^5^ p/s/cm^2^/sr.

### New CTZ analogues exert unique spectral separation

The BL spectral variance of the live cells stably expressing RLuc86SG or iRFP-RLuc86SG was examined in the presence of K5 or K6 (Fig. 4). The results interestingly showed that K5 had approximately 120-nm blue-shifted BL spectra with both RLuc86SG and iRFP-RLuc86SG (i.e., λ_max_ = 420 nm, compared with λ_max_ = 535 nm for native CTZ). On the other hand, K6 exerted only 55-nm blue-shifted BL spectra in the same condition (i.e., λ_max_ = 485 nm).

This strongly blue-shifted BL spectra indicate that three different colors (spectra) can be fabricated with RLuc86SG by simply replacing the substrate with native CTZ (535 nm), K6 (485 nm), or K5 (420 nm). We originally expected potential BRET signals with iRFP-RLuc86SG. We presume that the reduced BL intensity of iRFP-RLuc86SG compared to RLuc86SG is because the iRFP-RLuc86SG has less expression and folding efficiency, compared to RLuc86SG. No significant BRET signal was observed at near 700 nm with iRFP-RLuc86SG, that is because the BRET study was done in the absence of bilirubin as the fluorophore component of iRFP. This is a highly useful feature in multiplex bioassays with minimal optical cross-leakages between the reporter signals.

The annotations, 52- and 58-folds, in Fig. 4A means that RLuc86SG is 54-fold brighter with K5 than with K6 at 393 nm, whereas RLuc86SG is 58-fold brighter with K6 than with K5 at 523 nm. The spectral segregation is highly driven by the optical properties of the applied filters. If one uses optimal filters with narrower bandwidth, the segregation should be better.

Upon measurement of BL spectra, spectrometry generally requires much stronger light intensities than luminometer. Our spectrophotometer (AB-1850, ATTO) did not have enough sensitivity to measure reliable K2–ALuc16 spectra.

### The luciferase specificity and spectral separation enable the creation of multiplex quadruple assay systems with minimal optical cross-leakages between the reporter signals

To create such a multiplex assay system, every single-chain probe needs to be made of a distinctive kind of marine luciferase. Considering that there is no suitable RLuc-based single-chain probe, a unique RLuc-based single-chain probe were newly developed for application in a multiplex assay system (Suppl. Figure 2).

The RLuc-based probe, F-R86SG-F, was examined to determine if it rapamycin-dependently enhances the BL intensities in microslides (Suppl. Figure 2(A–C)). The right three channels stimulated with rapamycin exerted strong BL intensities, compared with the left three channels activated by the vehicle as control. The fold intensities were 3.1 and 1.6 with K6 and K5, respectively. Comparison of the absolute BL intensities showed that the overall BL intensities of the microslide illuminated with K6 were superior to those illuminated with K5.

### The multiplex quadruple bioluminescent assay system can simultaneously determine four distinctive ligands

Four different ligand activities were simultaneously determined with the multiplex quadruple bioluminescent assay system (Fig. 5). The results showed that K2 selectively illuminated only the cortisol-stimulated wells in the section, with 2.5-fold intensity. Similarly, K5 enhanced only the DHT-stimulated wells in the section, with 3.4-fold intensity. DBC did not elevate any considerable BL intensities in the same experimental condition. Interestingly, D-luciferin selectively illuminated both estrogen agonist (E_2_)- and antagonist (OHT)-stimulated wells in the section, with fold intensities of 5.3 and 3.9 (open filter), respectively.

Further examination was done to determine if the E_2_- and OHT-triggered BL images were influenced by 540-nm and 620-nm band-pass filters. It is because the 540 and 620 nm are the spectral peaks of CB green and CB red, respectively. The results showed that only the OHT-stimulated subsection was bright with a 620-nm band-pass filter. This indicated that the BL was solely contributed by the CB red probe, which was found to be sensitive only to OHT as an estrogen antagonist in our previous study^14^. On the other hand, both OHT- and E_2_-stimulated wells were found to luminesce in the presence of a 540-nm band-pass filter. This result corresponds with the previous conclusion that the CB green probe elevates the BL intensities by both OHT and E_2_^14^. The overall sensorial efficiency and S/N ratio may be further improved by optimization of the cotransfection ratios.

## Discussion

Luciferin is the energy source of BL and acts as “luminophore” in the BL-emitting reaction. Hence, luciferin is considered as the heart of the BL reaction^1^.

CTZ as a versatile marine luciferin has an imidazopyrazinon backbone, and its functional groups in the C-2, C-6, and C-8 positions have been targeted for better optical functionality^5,18,19^. In our previous study, the C-6 position was mainly targeted for the luciferase specificity of the CTZ analogues^12^.

Here, six new CTZ analogues were synthesized with modifications to the functional groups in the C-6 and C-8 positions. The new CTZ analogues are characterized by bulkiness and electron-donating properties, compared with native CTZ. To our knowledge, although many research groups have synthesized various CTZ analogues^5,18^, our approach is the first to study them in relation to multiple luciferase specificity.

It is difficult to decode the host–guest chemistry between marine luciferases and CTZ analogues.

We previously discussed that the luciferase specificity of CTZ analogues may be elucidated in the context of the quantitative chemical structure­bioluminescence intensity relationship (QSIR)^12^. For example, we previously concluded that: (i) the bulky functional groups at the C-6 position can be accommodated by ALucs, but relatively strict by RLuc, and (ii) the hydroxyphenyl group at the C-2 position positively acts for the specificity to ALuc.

The present study further found that the benzyl (or phenyl) group at the C-8 position is an important determinant for the marine luciferase specificity; i.e., the bulkiness at the C-8 position of K2 can be accommodated by ALuc series luciferases but not by RLuc series luciferases. This view explains the ALuc specificity of K2 and the RLuc specificity of K5 and K6. However, the explanation does not elucidate why ALucs are bright only with K2 but not with K1, K3, and K4, considering they are similar each other in the chemical structure. We presume that all K1–4 have basal potential to luminesce with ALucs, but K2 is clearly higher in BL intensity than the others.

In the present work, significant autoluminescence was observed when K6 was mixed with FBS. The intentional significancy is followed by K3 (Fig. S1).

The discussion of the origin of K6-driven autoluminescence in serum is intriguing. Several studies have concluded that: (i) serum albumin occupies ca. 55% of serum proteins in the human body^20^, (ii) albumin can interact with small organic compounds^21^, and (iii) albumin can catalyze CTZ chemiluminescence consistent with a monooxygenase^22^. These references suggest that the K6-driven autoluminescence is greatly contributed by albumin in the serum.

This view is further supported by a study by Nishihara et al.^23^, in which an albumin-specific CTZ analogue named HuLumino1 was synthesized. K6 shares a similar chemical structure with HuLumino1 in that: (i) the functional group at the C-8 position is commonly eliminated, and (ii) the *p*-position of the C-6 is commonly modified with a weak electron-donating group. The corresponding molecular modeling of Nishihara et al. also shows that the hydrophobic site of albumin interacts with HuLumino1 through π-electron stacking. Further, we hypothesize that K6 binds serum albumin in the hydrophobic pocket and is catalytically decomposed. On the other hand, it still remains unclear why the autoluminescence of K5 is not as strong as that of K6 in serum, although K5 is chemically similar to K6 and HuLumino1.

A comparison of the chemical structures of K2 and K5 is also interesting toward understanding the reason behind their specificity to ALuc and RLuc, respectively. The chemical structural comparison shows that: (i) the bulky phenyl group at the C-8 position is essential for ALuc derivatives, although it may negatively work for RLuc derivatives; (ii) on comparison of the structures of K2, K5, and K6, the dimethylaminophenyl group at the C-6 position is not necessarily required for RLuc specificity and is replaceable with the benzodioxane group; (iii) a comparison of the structures of K2 and K4 indicates that both the dimethylaminophenyl and the benzodioxane group are necessary for the ALuc specificity; and (iv) the introduction of any bulky functional group to the C-8 position always works negatively for the RLuc activity.

Moreover, it is interesting to reason the blue-shifted BL spectra of K5 and K6, compared with that of nCTZ. In our measurement, the spectral peak (λ_max_) of RLuc86SG with nCTZ was determined at 535 nm. However, the λ_max_ is 55-nm blue-shifted with K6 and further blue-shifted up to 115 nm with K5, compared with nCTZ.

It is interpreted that the blue-shifts in the λ_max_ values are highly contributed by the shortened π-electron conjugation in the structures of K5 and K6, considering that they are deficient in the functional groups at the C-8 position. Additionally, the more blue-shifted λ_max_ values of K5 than those of K6 may indicate that the benzodioxane group of K5 has less π-electron donating effect than the dimethylaminophenyl group, which may contribute to the shortened π-electron conjugation.

Regarding the blue-shifted spectra of CTZ analogues, Shimomura et al. explored the reason with the presence or absence of the *p*-hydroxyl group at the C-6 position in the CTZ analogues^24^. They explained that the *p*-hydroxyl group at the C-6 position of native CTZ forms the pyrazine anion on enzymatic oxidation. Thus, the absence of the *p*-hydroxyl group, as in the K-series CTZ analogues in this work, cannot exert such a pyrazine anion and consequently resulted in BL emission at shorter wavelengths.

Spectral overlaps among the BL spectra of marine luciferases, a common drawback in multiplex assays, need to be addressed. This is because such optical cross-leakage impairs multiplex imaging in bioassays. To minimize optical contamination, tactical algorithms for unmixing the overlapped spectra have been developed^9^. However, these have not provided a fundamental solution to solve the problem. On the other hand, the 115-nm and 55-nm blue-shifted spectra of K5 and K6 significantly minimize optical contamination in multiplex assay systems. The natural spectral separation of the BL signals contributes considerably to the signal separation efficiency with optical filters in multiplex assays.

Multiplex assay systems have great merit in the specific and high throughput determination of multiple samples. In this study, the overall luciferase specificity and blue-shifted features of K-series CTZ analogues encouraged the creation of a multiplex quadruple bioluminescent assay system for simultaneous assaying of multiple ligands in a mixture sample. Four distinctive luciferase-based single-chain probes were genetically integrated into a set of mammalian expression vectors for constituting the multiplex quadruple bioluminescent assay system. This application enabled the selective and quantitative imaging of multiple ligand activity simultaneously with the specific substrates.

The assay system is designed to express four different single-chain probes simultaneously, each of which specifically determines its own ligand (first authentication). The only ligand-activated single-chain probe can be illuminated with specific substrates, such as K2, K5, or D-luciferin (second authentication). Thus, through this two-step authentication, the assay system minimizes false positive signals.

The working mechanism with two authentication points may be also explained with two “on–off” switches (i.e., Ligand and Substrate), which are enough to overcome the insufficient separation peaks of Fig. 4: The first on–off switch is the ligand, which stimulates only its specific probe in the quadruple system. All the other probes remain inactivated. The ligand-stimulated probe alone in the quadruple system is eligible to emit the BL signal only when the specific substrate exists as the second on–off switch.

This quadruple system is unique that all the probes’ signals remain inactivated in the absence of a specific ligand: it is a common merit of “protein-fragment complementation assay (PCA)” probes we used, in which the embedded luciferases inside the probes are designed to be fragmented and thus temporarily inactivated in the absence of the specific ligand.

This study showed how the multiplex assay system can be designed to determine multiple steroid hormones or chemicals. It is generally difficult for conventional bioassays to simultaneously determine multiple steroid hormones or chemicals in a mixture. This is because steroid hormones for example share similar chemical structures and activate their corresponding nuclear receptors (NRs) through complex intracellular signal transduction pathways in cells^25^. Nevertheless, in the present work, four distinctive ligands were specifically determined by using specific CTZ analogues and unique single-chain probes in the multiplex assay system.

The workflow of the quadruple system is very simple and labor-effective. No washing nor quenching steps are necessary in the system. The cells containing the four probes are firstly stimulated with each ligand in 96-well microplates. The wells are then conceptionally divided into four sections, each of which are illuminated by an injection of the specific substrates, K2, K5, DBC, or D-luciferin after elimination of the medium. This assay protocol is highly convenient, compared to conventional dual-luciferase assay systems, which require quenching and/or washing steps. In addition, as explained in the workflow, every single well on the 96-well microplate is luminesced by a different specific substrate, where each well is not luminesced by multiple substrates. It is thus impossible that the spectral contamination among the BLs occurs in wells.

This multiplex assay system is supported by (i) the novel CTZ analogues, that are exclusively sensitive to specific luciferases and by (ii) a single lineage of the multi-sensorial cells. Hence, all the cell-seeded wells have completely equivalent sensorial properties to ligands. Moreover, all the wells can be “simultaneously” imaged by one-shot injection of multiple substrate solutions with 12-channel pipettes.

Upon substrate injection, the wells may be illuminated with two optional methods: i.e., option (1) is to add single substrate to each well as shown in Fig. 5; option (2) is to add multiple substrates in cocktail to each well on the microplate. In the case of option (2), the substrate cocktail may cause a potential cross-leakage of the signals. In the present study, the option (1) is better to accomplish our study goals.

When the option (1) is applied for the signal development, the potential contribution of the cross-leakage in the optical signals was addressed in Fig. 3. Thanks to the quantitative specificity data set of the substrates in Fig. 3, one should assume the extent of the cross-leakage in the total optical signal, where the background signals (0%-contents) were also examined.

In summary, this research newly synthesized a series of CTZ analogues, named the K-series, and achieved the selective illumination of luciferases, which was applied to specifically visualize only one ligand-activated single-chain bioluminescent probe among many. In addition, the K-series CTZ analogues were found to exert blue-shifted BL spectra with RLuc series luciferases. The luciferase specificity and color variation of the CTZ analogues minimized optical signal cross-leakages and enabled screening of specific ligands in the mixture. The specificity of the substrates was further tailored to marine luciferases (or single-chain bioluminescent probes) to create a multiplex quadruple assay system containing four single-chain bioluminescent probes. Each probe is able to luminesce only in the combined presence of a specific CTZ analogue and a specific ligand in the multiplex quadruple assay system. This unique multiplex quadruple bioluminescent assay system is a potential optical platform for specific and high throughput imaging of multiple optical markers in bioassays without optical cross-leakages.

## Supplementary Information

Supplementary Information.

## Data Availability

The datasets used and/or analysed during the current study available from the corresponding author on reasonable request.
