# Supplementary material for: Multiplex quadruple bioluminescent assay system
Source: Sci Rep. 2022 Oct 19;12:17485. doi: 10.1038/s41598-022-20468-1 (PMC9581999; doi:10.1038/s41598-022-20468-1)
Supplement: Supplementary file 1 — Supplementary Information. [file 41598_2022_20468_MOESM1_ESM.docx]

Suppl. Information

Multiplex quadruple bioluminescent assay system

Genta Kamiya,^a^ Nobuo Kitada,^a^ Shojiro Maki,^a,^* Sung Bae Kim, ^b,^*

a. Department of Engineering Science, Graduate School of Informatics and Engineering, The University of Electro-Communications, Chofu, Tokyo, 182-8585, Japan

b. Research Institute for Environmental Management Technology, National Institute of Advanced Industrial Science and Technology (AIST), 16-1 Onogawa, Tsukuba 305-8569, Japan.

**Contents**

Suppl. Figures page 2

Suppl. Experimental Procedure page 4

**Suppl. Figures**


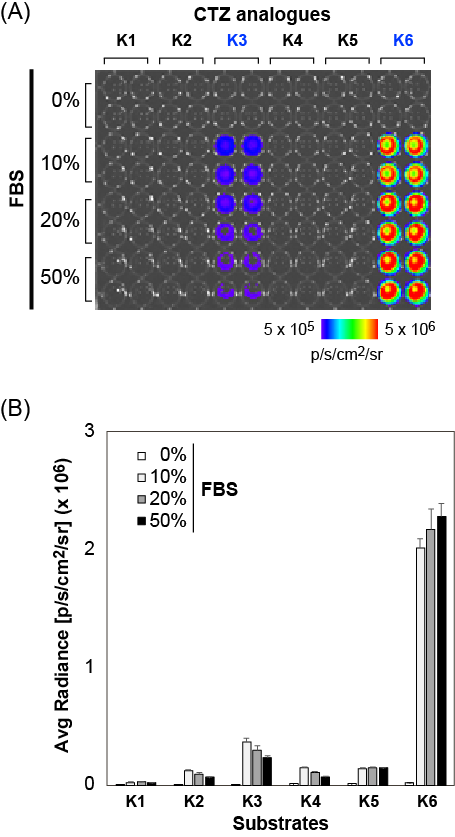


**Suppl Figure 1**. Determination of the autoluminescence of the K-series CTZ analogues with varying ratios of FBS. **(A)** BL image showing significant autoluminescence. **(B)** The corresponding absolute intensities of the BL image.


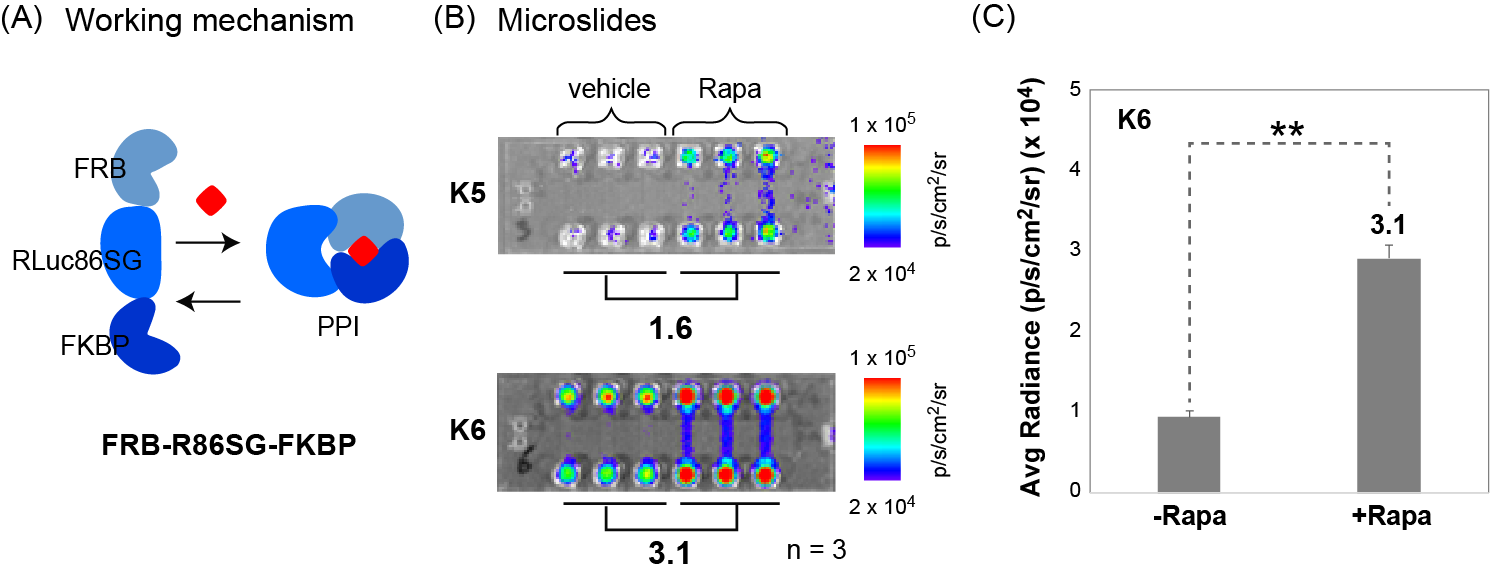


**Suppl. Figure 2.** Development of new single-chain bioluminescent probes carrying RLuc86SG. **(A)** Schematic illustration of the working mechanism of a single-chain molecular strain probe. RLuc86SG is sandwiched between FRB and FKBP. Rapamycin triggers intra- and/or intermolecular protein―protein interactions. This conformation change enhances BL intensities. **(B)** The K5- and K6-driven BL images of the probe FRB-R86SG-FKBP in the presence or absence of rapamycin on the microslides. **(C)** The corresponding absolute BL intensities with and without rapamycin.

**Suppl. Experimental Procedure**

**1.1 General procedure of the organic synthesis**

Commercially available reagents and solvents were used without further purification. Silica gel 70 plates (F254 TLC, Wako) were used for analytical TLC, and Chemical Silica gel 60 N plates (spherical, neutral, Kanto) were used for column chromatography. For preparative flash chromatography, an automated system (Smart Flash EPCLC AI-580S, Yamazen Corp., Japan) were used with universal columns of silica gel. ^1^Hand ^13^C NMR spectra were recorded on a ECA-500 instrument (JEOL) (500 MHz for ^1^H and 126 MHz for ^13^C). BLspectra was measured with a spectrophotometer (ATTO AB-1850) with a 1-nm data interval. BLI was determined with a multifunctional in vivo imaging system (IVIS Spectrum, PerkinElmer).

The CTZ analogues **K1-K5** were synthesized according to the following scheme (Suppl. Figure 3).

We conducted a Suzuki-Miyaura coupling reaction using 2-amino-3,5-dibromoaminopyrazine **1** and 4-(dimethylamino) phenylboronic Acid or 1,4-Benzodioxane-6-boronic Acid to produce 5-bromo-3-(4-(dimethylamino) phenyl) pyrazine-2-anino **4** and 5-bromo-3-(2,3-dihydrobenzo [b] [1,4] dioxin-6-yl)pyrazin-2-amine **5**. The made **4** and **5** were additionally reacted with 4-(dimethylamino) phenylboronic acid or 1,4-Benzodioxane-6-boronic Acid through Suzuki-Miyaura coupling to create the aminopyrazine derivatives **6-9**.

Likewise, we conducted a Suzuki-Miyaura coupling reaction using 2-amino-5-dibromoaminopyrazine **2** and 4-(dimethylamino) phenylboronic Acid or 1,4-Benzodioxane-6-boronic Acid to produce the aminopyrazine derivatives **10** and **11.**

Finally, the synthesized aminopyrazine derivatives **6-11** reacted with the ketoacetal derivatives **12** prepared by the Grignard reaction, and condensed and cyclized under hydrochloric acid conditions.

The synthesized CTZ analogues were named **K1-K6**, respectively.


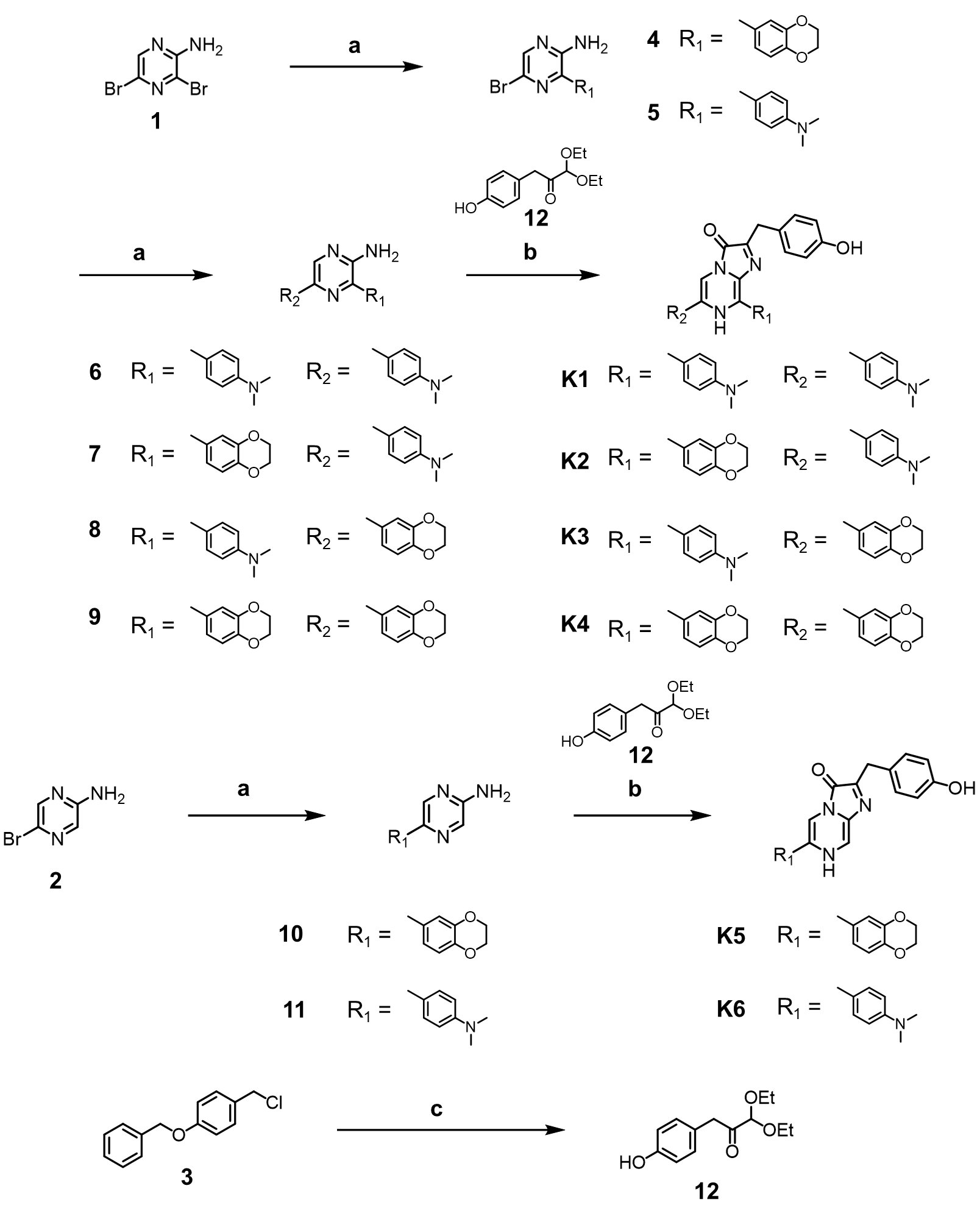


**Suppl. Figure 3.** Synthetic scheme for K1-K6. a) Pd(PPh_3_)_4_, 2M NaCO_3_, 1,4-Dioxane; b) 12M HCl, EtOH; c) ⅰ) Mg, (EtO)_2_AcOEt, THF, Ar ⅱ)Pd / C, EtOH, H_2_


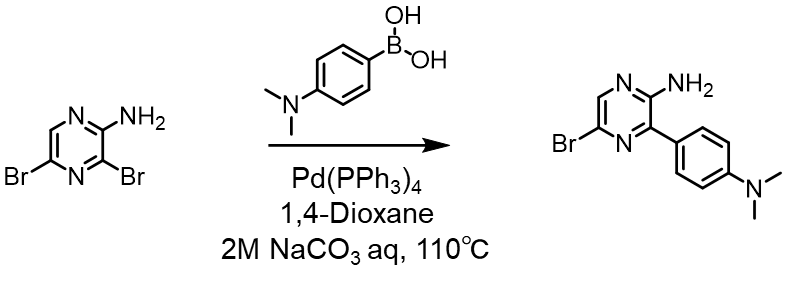


**5**

**1**

**1.2 The specific procedure of the organic synthesis and the corresponding NMR data of the K-series CTZ analogues.**

**5-bromo-3-(2,3-dihydrobenzo [b] [1,4] dioxin-6-yl)pyrazin-2-amine** **
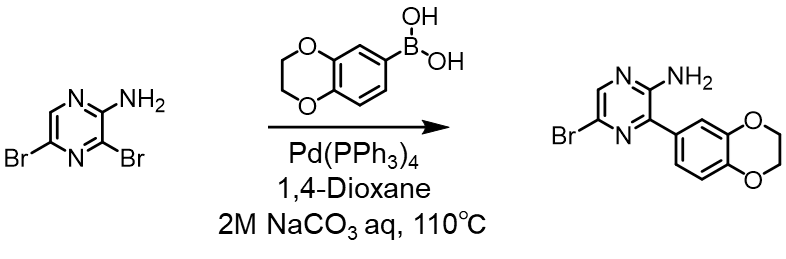
4**

**4**

**1**

2-Amino-3,5-dibromopyrazine **1** (100 mg, 0.395 mmol), (1,4-Benzodioxan-6-yl)-boronic acid (78.19 mg, 0.435 mmol), tetrakis(triphenylphosphine)palladium (22 mg, 0.019 mol), was dissolved in 2 mL of 1,4-dioxane. 2 mL of a 2 M aqueous solution of sodium carbonate was added under argon atmosphere, and the mixture was heated under reflux at 110 ° C for 4 hours. After the reaction was completed, 30 mL of water was added, and the product was extracted with ethyl acetate (2 × 40 mL). The organic phase was washed with saturated saline, dried over anhydrous sodium sulfate, and then concentrated under reduced pressure. The obtained yellow solid substance was separated by column chromatography (hexane / ethyl acetate = 1/1 to ethyl acetate) to obtain a compound **4** (95 mg, 0.311mmol, 78%) as a yellow crystal.

^1^H-NMR (500 MHz, CHLOROFORM-D) δ 8.01 (s, 1H), 7.25 (d, *J* = 1.7 Hz, 1H), 7.21 (dd, *J* = 8.6, 2.3 Hz, 1H), 6.96 (d, *J* = 8.6 Hz, 1H), 4.79 (s, 2H), 4.31-4.27 (m, 4H)

^13^C-NMR (126 MHz, CHLOROFORM-D) *δ* 151.2, 144.9, 144.1, 142.4, 140.4, 129.2, 127.0, 121.4, 118.1, 117.4, 64.7, 64.4

HR-ESI-MS: *m/z*: [M+H]^+^ calcd for C_12_H_11_Br_1_N_3_O_2_, 308.00366; found, 308.00346

Compounds **5-11** were prepared with a similar procedure to the preparation of **4**.

**5-bromo-3-(4-(dimethylamino) phenyl) pyrazine-2-anino** **5** (95 mg, 0.325 mmol, 82%); yellow crystals.

^1^H-NMR (500 MHz, CHLOROFORM-D) δ 7.95 (s, 1H), 7.64 (d, *J* = 9.2 Hz, 2H), 6.77 (d, *J* = 8.6 Hz, 2H), 4.76 (s, 2H), 3.02 (s, 6H)^13^C-NMR (126 MHz, CHLOROFORM-D) δ 151.2, 151.2, 141.9, 141.1, 129.2, 127.2, 123.3, 112.3, 40.4

HR-ESI-MS: *m/z*: [M+H]^+^ calcd for C_12_H_14_Br_1_N_4_, 293.04197; found, 293.04018


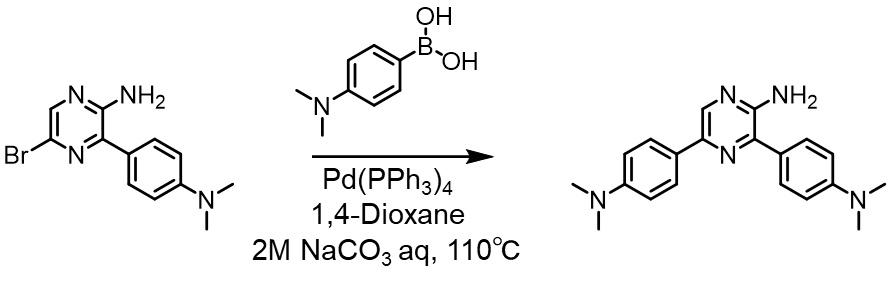


**6**

**5**

**4,4'-(3-aminopyrazine-2,6-diyl)bis(N,N-dimethylaniline) 6** (123 mg, 0.370 mmol, 72%); green crystals

^1^H-NMR (500 MHz, CHLOROFORM-D) δ 8.28 (s, 1H), 7.87 (dd, *J* = 6.9, 2.3 Hz, 2H), 7.76 (d, *J* = 9.2 Hz, 2H), 6.82 (d, *J* = 8.6 Hz, 2H), 6.78 (d, *J* = 9.2 Hz, 2H), 4.69 (s, 2H), 3.02 (s, 6H), 2.99 (s, 6H)

^13^C-NMR (126 MHz, CHLOROFORM-D) δ 150.9, 150.6, 150.0, 135.3, 132.3, 132.2, 129.4, 128.7, 128.6, 126.7, 112.7, 112.4, 40.7, 40.5


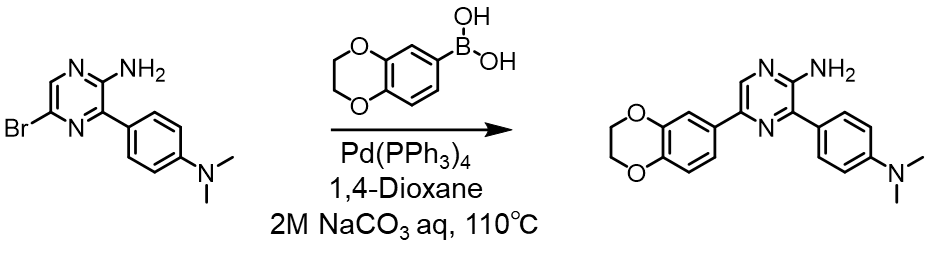
HR-ESI-MS: *m/z*: [M+H]^+^ calcd for C_20_H_24_N_5_, 334.20400; found, 334.20317

**7**

**5**

**5-(2,3-dihydrobenzo[b][1,4]dioxin-6-yl)-3-(4-(dimethylamino)phenyl)pyrazin-2-amine 7** (89 mg, 0.256 mmol, 74%) as a yellow crystals

^1^H-NMR (500 MHz, CHLOROFORM-D) δ 8.26 (s, 1H), 7.75 (d, *J* = 8.6 Hz, 2H), 7.52 (d, *J* = 1.7 Hz, 1H), 7.46 (dd, *J* = 8.6, 2.3 Hz, 1H), 6.91 (d, *J* = 8.6 Hz, 1H), 6.81 (d, *J* = 8.6 Hz, 2H), 4.74 (s, 1H), 4.29 (s, 4H), 3.03 (s, 6H)

^13^C-NMR (126 MHz, CHLOROFORM-D) δ 150.9, 150.5, 135.7, 132.1, 132.1, 131.9, 131.9, 129.3, 128.5, 128.5, 118.9, 117.4, 114.7, 112.3, 64.5, 64.4, 40.4

HR-ESI-MS: *m/z*: [M+H]^+^ calcd for C_20_H_21_N_4_O_2_, 349.16618; found, 349.16645


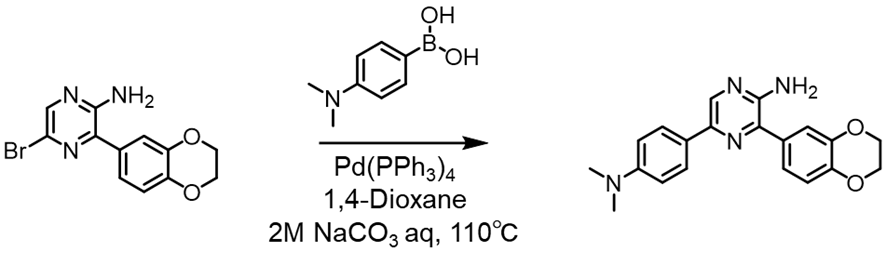


**8**

**4**

**3-(2,3-dihydrobenzo[b][1,4]dioxin-6-yl)-5-(4-(dimethylamino)phenyl)pyrazin-2-amine 8** (95 mg, 0.273 mmol, 84%) as a yellow crystals

^1^H-NMR (500 MHz, CHLOROFORM-D) δ 8.33 (s, 1H), 7.86 (dd, *J* = 6.9, 1.7 Hz, 2H), 7.38 (d, *J* = 2.3 Hz, 1H), 7.33 (dd, *J* = 8.3, 2.0 Hz, 1H), 6.98 (d, *J* = 8.0 Hz, 1H), 6.78 (d, *J*= 8.6 Hz, 2H), 4.66 (s, 2H), 4.31 (s, 4H), 2.99 (s, 6H)

^13^C-NMR (126 MHz, CHLOROFORM-D) δ 150.7, 149.9, 144.4, 144.0, 143.8, 138.8, 136.4, 131.3, 126.7, 125.5, 121.6, 117.8, 117.7, 112.6, 64.7, 64.5, 40.6

HR-ESI-MS: *m/z*: [M+H]^+^ calcd for C_20_H_21_N_4_O_2_, 349.16742; found, 349.16645


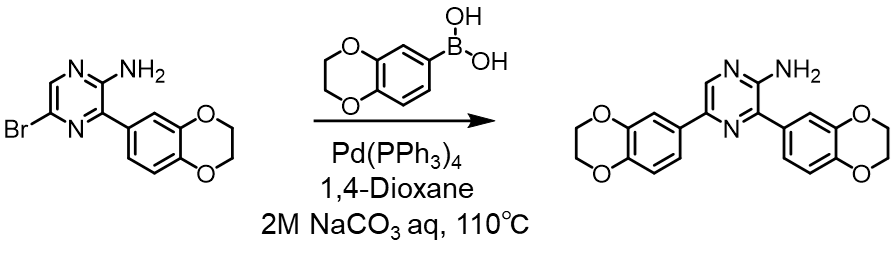


**4**

**9**

**3,5-bis(2,3-dihydrobenzo[b][1,4]dioxin-6-yl)pyrazin-2-amine 9** (154 mg, 0.325 mmol, 87%) as a yellow crystals

^1^H-NMR (500 MHz, CHLOROFORM-D) δ 8.31 (s, 1H), 7.50 (d, *J* = 2.3 Hz, 1H), 7.46-7.43 (m, 1H), 7.36 (d, *J* = 1.7 Hz, 1H), 7.34-7.31 (m, 1H), 6.98 (t, *J* = 4.3 Hz, 1H), 6.91 (d, *J* = 8.0 Hz, 1H), 4.76 (s, 2H), 4.32-4.29 (m, 8H)

^13^C-NMR (126 MHz, CHLOROFORM-D) δ 150.5, 144.5, 144.0, 143.9, 143.9, 142.6, 139.0, 136.9, 131.0, 131.0, 121.6, 119.1, 117.9, 117.6, 117.3, 114.8, 64.7, 64.5

HR-ESI-MS: *m/z*: [M+H]^+^ calcd for C_20_H_18_N_3_O_4_, 364.13046; found, 364.12973


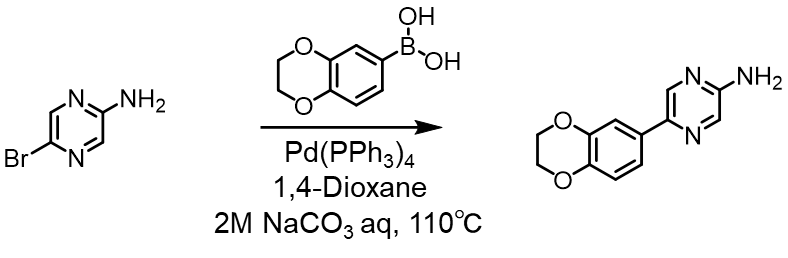


**10**

**2**

5-(2,3-dihydrobenzo[b][1,4]dioxin-6-yl)pyrazin-2-amine 10 (213 mg, 0.931 mmol, 81%) as a yellow crystals

1H-NMR (500 MHz, CHLOROFORM-D) δ 8.36 (d, J = 1.7 Hz, 1H), 8.02 (d, J = 1.1 Hz, 1H), 7.40 (d, J = 2.3 Hz, 1H), 7.36 (dd, J = 8.6, 2.3 Hz, 1H), 6.93 (d, J = 8.6 Hz, 1H), 4.52 (s, 2H), 4.29 (s, 4H)

13C-NMR (126 MHz, CHLOROFORM-D) δ 152.8, 144.0, 144.0, 142.9, 138.7, 131.5, 130.9, 119.0, 117.8, 114.8, 64.7, 64.5

HR-ESI-MS: *m/z*: [M+H]^+^ calcd for C_12_H_12_N_3_O_2_, 230.09494; found, 230.09295


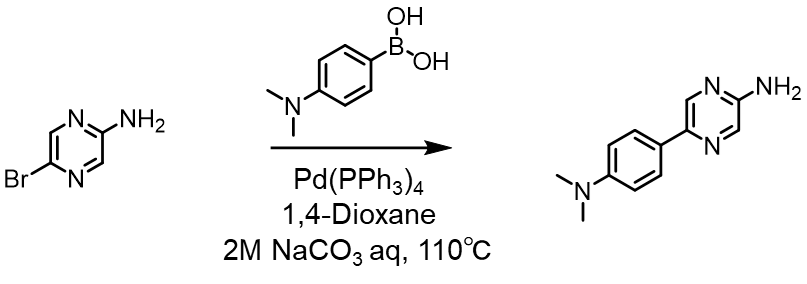


**11**

**2**

**5-(4-(dimethylamino)phenyl)pyrazin-2-amine** **11** (213 mg, 0.994 mmol, 87%) as a yellow crystals

1H-NMR (500 MHz, CHLOROFORM-D) δ 8.37 (d, J = 1.1 Hz, 1H), 8.01 (d, J = 1.1 Hz, 1H), 7.76 (td, J = 6.0, 3.4 Hz, 2H), 6.79 (td, J = 6.0, 3.4 Hz, 2H), 4.46 (s, 2H), 3.00 (s, 6H)

13C-NMR (126 MHz, CHLOROFORM-D) δ 152.2, 150.7, 143.9, 138.1, 131.4, 126.7, 125.3, 112.7, 40.6

HR-ESI-MS: *m/z*: [M+H]^+^ calcd for C_12_H_15_N_4_, 215.13097; found, 215.12964


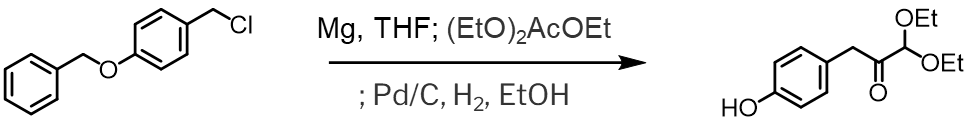


**3**

**12**

**1,1-diethoxy-3-(4-hydroxyphenyl) propan-2-one 12**

Mg turnings (666 mg, 27.50 mmol,) and 1,2-Dibromoethane (0.2 mL) were added to THF (10 ml) and stirred under argon atmosphere for 20 minutes. solution of 4-(Benzyloxy) benzyl Chloride **3** (1.6 g, 6.88 mmol) in THF (10 mL) was added and the mixture was stirred at r.t. for 30 min. Then mixture was further refluxed for 1.5 h to complete the reaction. The Grignard reagent was lowed to cool to r.t. and was then kept in an ice bath. Ethyl diethoxyacetate (1.29 ml, 7.28 mmol) was dissolved in THF (10 mL) under an argon atmosphere and cooled to –78 °C. The Grignard reagent was transferred dropwise into the cooled flask over 15 min. The mixture was then stirred at –78 °C for 2 h. After the reaction was completed, 70 mL of water was added, and the product was extracted with ethyl acetate (3 × 100 mL). The organic phase was washed with saturated saline, dried over anhydrous sodium sulfate, and then concentrated under reduced pressure.

The obtained white oil substance was dissolved in MeOH (50 mL) and 10% Pd/C (190 mg) was added, and the mixture was placed under H_2_ gas. The compound was stirred for 24 hours and then filtered through Celite, and then concentrated under reduced pressure. The white oil substance was separated by column chromatography (hexane / ethyl acetate = 5 / 1 → 4 / 1) to obtain compound **12** (665 mg, 2.79 mol, 40%) as a colorless oil.

^1^H-NMR (500 MHz, CHLOROFORM-D) δ 7.05 (dd, J = 11.5, 2.9 Hz, 2H), 6.75 (dd, J = 11.5, 3.4 Hz, 2H), 5.58 (s, 1H), 4.65 (s, 1H), 3.82 (s, 2H), 3.73-3.67 (m, 2H), 3.58-3.52 (m, 2H), 1.24 (t, J = 7.2 Hz, 6H)

^13^C-NMR (126 MHz, CHLOROFORM-D) δ 204.5, 154.9, 130.9, 125.3, 115.6, 102.1, 63.5, 43.1, 15.2

HR-ESI-MS: *m/z*: [M+Na]^+^ calcd for C_13_H_18_Na_1_O_4_, 261.11141; found, 261.11028


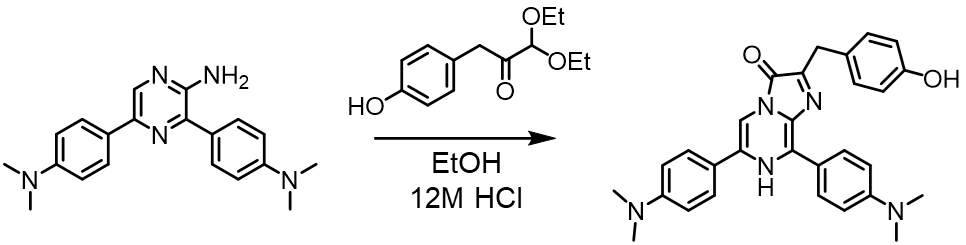


**6**

**K1**

**6,8-bis(4-(dimethylamino)phenyl)-2-(4-hydroxybenzyl)imidazo[1,2-a]pyrazin-3(7H)-one K1**

The aminopyrazine derivative **6** (30 mg, 0.083 mmol) and ketoacetal **12** (29 mg, 0.124 mmol) were dissolved in ethanol (2.0 mL), 12 M hydrochloric acid (100 μL) was added, and the mixture was stirred at 65° C. for 12 hours. After completion of the reaction, the reaction mixture was concentrated under reduced pressure. The obtained residue was separated by automated flash chromatography (Chloroform / Methanol) to give CTZ analog **K1** (17 mg, 0.035 mmol, 21%) as a red crystals.

^1^H-NMR (500 MHz, METHANOL-D3) δ 8.94 (s, 1H), 8.41 (d, J = 8.6 Hz, 2H), 8.22 (d, J = 9.2 Hz, 2H), 7.90 (d, J = 9.2 Hz, 2H), 7.69 (d, J = 8.6 Hz, 2H), 7.14 (d, J = 8.6 Hz, 2H), 6.74 (d, J = 8.6 Hz, 2H), 4.25 (s, 2H), 3.37 (s, 6H), 3.32 (s, 6H)

HR-ESI-MS: *m/z*: [M+H]^+^ calcd for C_29_H_30_N_5_O_2_, 480.24277; found, 480.23995

Compounds **K2-K5** were prepared with a similar procedure to the preparation of **4**.


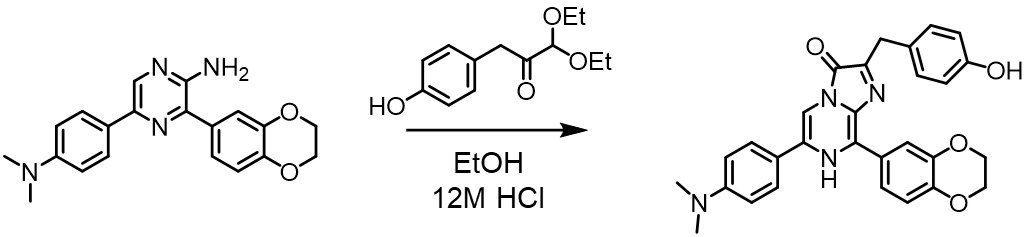


**K2**

**7**

**8-(2,3-dihydrobenzo[b][1,4]dioxin-6-yl)-6-(4-(dimethylamino)phenyl)-2-(4-hydroxybenzyl)imidazo[1,2-a]pyrazin-3(7H)-one K2** (20 mg, 0.041 mmol, 47%) as a red crystals.

^1^H-NMR (500 MHz, METHANOL-D4) δ 8.77 (s, 1H), 8.30 (d, J = 8.6 Hz, 2H), 7.76 (d, J = 8.6 Hz, 2H), 7.53 (td, J = 7.7, 2.3 Hz, 2H), 7.13 (d, J = 8.6 Hz, 2H), 7.05 (d, J = 8.6 Hz, 1H), 6.74 (d, J = 8.0 Hz, 2H), 4.34-4.29 (m, 4H), 4.21 (s, 2H), 3.32-3.36 (s, 6H)

HR-ESI-MS: *m/z*: [M+H]^+^ calcd for C_29_H_27_N_4_O_4_, 495.20262; found, 495.20323


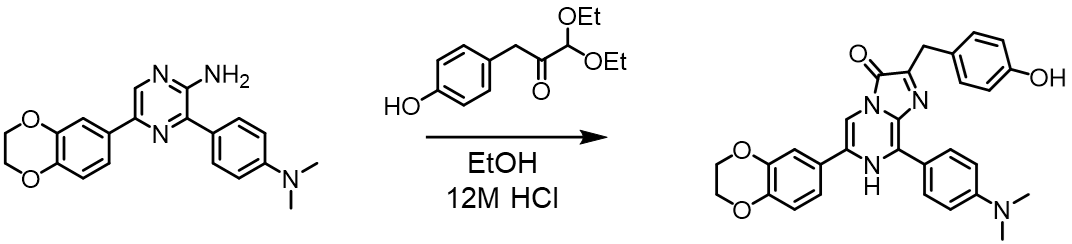


**8**

**K3**

**6-(2,3-dihydrobenzo[b][1,4]dioxin-6-yl)-8-(4-(dimethylamino)phenyl)-2-(4-hydroxybenzyl)imidazo[1,2-a]pyrazin-3(7H)-one K3** (24 mg, 0.048 mmol, 56%) as a red crystals.

^1^H-NMR (500 MHz, METHANOL-D3) δ 8.57 (s, 1H), 8.18 (d, J = 8.6 Hz, 2H), 7.68 (d, J = 8.6 Hz, 2H), 7.56 (d, J = 1.7 Hz, 1H), 7.52 (dd, J = 8.3, 2.0 Hz, 1H), 7.12 (d, J = 8.0 Hz, 2H), 6.95 (d, J = 8.0 Hz, 1H), 6.72 (d, J = 8.6 Hz, 2H), 4.28 (s, 4H), 4.20 (s, 2H), 3.30 (s, 6H)

HR-ESI-MS: *m/z*: [M+H]^+^ calcd for C_29_H_27_N_4_O_4_, 495.20188; found, 495.20323


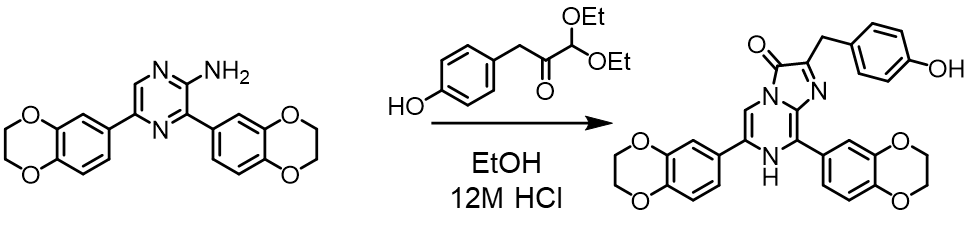


**9**

**K4**

**6,8-bis(2,3-dihydrobenzo[b][1,4]dioxin-6-yl)-2-(4-hydroxybenzyl)imidazo[1,2-a]pyrazin-3(7H)-one K4**

CTZ analog **K4** (21 mg, 0.041 mmol, 49%) as a red crystals.

^1^H-NMR (500 MHz, METHANOL-D3) δ 8.31 (s, 1H), 7.57 (d, J = 1.7 Hz, 1H), 7.52 (dd, J = 8.6, 2.3 Hz, 1H), 7.51 (d, J = 2.3 Hz, 1H), 7.46 (dd, J = 8.3, 2.0 Hz, 1H), 7.10 (d, J = 8.6 Hz, 2H), 7.06 (d, J = 8.0 Hz, 1H), 6.96 (d, J = 8.0 Hz, 1H), 6.70 (dd, J = 6.6, 2.0 Hz, 2H), 4.35-4.28 (m, 8H), 4.13 (s, 2H)

HR-ESI-MS: *m/z*: [M+H]^+^ calcd for C_29_H_24_N_3_O_6_, 510.16722; found, 510.16651


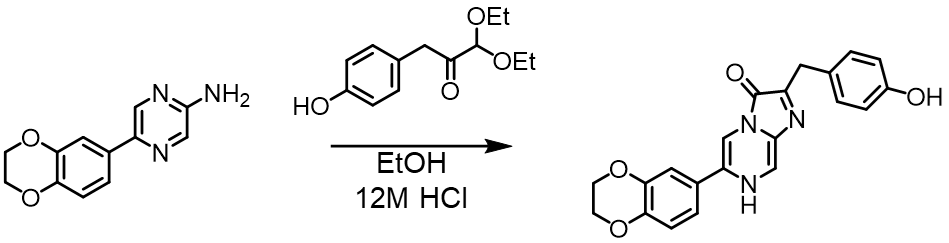


**10**

**K5**

**6-(2,3-dihydrobenzo[b][1,4]dioxin-6-yl)-2-(4-hydroxybenzyl)imidazo[1,2-a]pyrazin-3(7H)-one K5** (17 mg, 0.045 mmol, 34 %) as a brown crystals.

^1^H-NMR (500 MHz, METHANOL-D3) δ 7.88 (s, 1H), 7.65 (s, 1H), 7.17 (d, J = 2.3 Hz, 1H), 7.14-7.11 (m, 3H), 6.93 (d, J = 8.0 Hz, 1H), 6.67 (dt, J = 9.2, 2.4 Hz, 2H), 4.26 (s, 4H), 4.02 (s, 2H)

HR-ESI-MS: *m/z*: [M+H]^+^ calcd for C_21_H_18_N_3_O_4_, 376.13145; found, 376.12973

**11**

**K6**


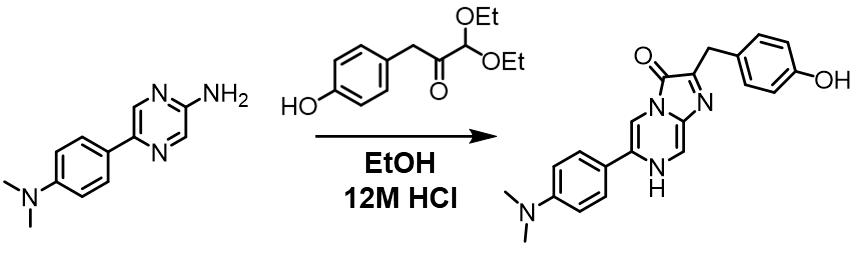


**6-(4-(dimethylamino)phenyl)-2-(4-hydroxybenzyl)imidazo[1,2-a]pyrazin-3(7H)-one K6** (34 mg, 0.094 mmol, 67%) as a brown crystals.

^1^H-NMR (500 MHz, METHANOL-D4) δ 7.86 (s, 1H), 7.60 (s, 1H), 7.49 (d, J = 8.6 Hz, 2H), 7.13 (d, J = 8.6 Hz, 2H), 6.82 (d, J = 8.6 Hz, 2H), 6.68 (d, J = 8.6 Hz, 2H), 4.02 (s, 2H), 2.99 (s, 6H)

HR-ESI-MS: *m/z*: [M+H]^+^ calcd for C_21_H_21_N_4_O_2_, 361.16690; found, 361.16645
